# Effect of Nrf2 Activators in Hepatitis B Virus-Infected Cells Under Oxidative Stress

**DOI:** 10.3390/md23040155

**Published:** 2025-04-03

**Authors:** Junsei Taira, Takuya Kubo, Hiroya Nagano, Ryuji Tsuda, Takayuki Ogi, Kenji Nakashima, Tetsuro Suzuki

**Affiliations:** 1 Department of Bioresources Technology, National Institute of Technology, Okinawa College, Okinawa 905-2192, Japans2321416@u.tsukuba.ac.jp (R.T.); 2 Department of Environment and Natural Resources, Okinawa Industrial Technology Center, Okinawa 904-2234, Japan; 3 Department of Microbiology and Immunology, Hamamatsu University School of Medicine, Hamamatsu 431-3192, Japan; kenjin@hama-med.ac.jp (K.N.); tesuzuki@hama-med.ac.jp (T.S.)

**Keywords:** oxidative stress, HBV (hepatitis B virus), Nrf2/ARE signaling, fucoxanthin, pteryxin, pregenomic RNA

## Abstract

The liver is an active metabolic site that generates high levels of reactive oxygen species (ROS). Oxidative stress has been implicated in the chronicity of hepatitis and hepatitis B virus (HBV) infection. This study aimed to determine the involvement of oxidative stress in HBV-infected cells and the efficacy of natural Nrf2 activators. The intracellular HBV pregenomic RNA copy number relative to total RNA was measured by RT-PCR, and various protein expressions associated with oxidative stress were analyzed by a Western blot analysis. The results showed that the Nrf2, HO-1, Akt, and Bcl-xL proteins were decreased by the continuous infection, indicating that HBV-positive cells were exposed to oxidative stress. The present study evaluated the anti-HBV infection effects of the Nrf2 activator fucoxanthin (Fx), a marine carotenoid from edible biological resources, including the comparative natural Nrf2 activator pteryxin (Ptx). These Nrf2 activators suppressed the HBV pregenomic RNA production in the HBV-infected cells, thus increasing the expression of the proteins of Nrf2 and HO-1. In the persistently infected cells transfected with the HBV genome, the Bcl-xL and Keap1 proteins, which contribute to suppressing the HBx protein involved in the HBV replication, were overexpressed. In particular, the activity of these protein expressions was marked at low concentrations of Fx. This suggests that natural Nrf2 activators may play a significant role in the HBV infection and could be a valuable source for further development through the functional utilization of food resources.

## 1. Introduction

Hepatitis B virus (HBV), belonging to the Hepadnaviridae family, is a virus with a double-stranded circular DNA genome of 3.2 kilobases in length. In 2019, approximately 296 million people, despite the availability of an effective prophylactic vaccine, were HBV-infected worldwide, and a total of 1.5 million people are annually infected [1]. HBV migrates into the nucleus and replicates itself via reverse transcription of the pregenomic RNA with a specific initiation mechanism. It then expresses the viral protein, hepatitis B virus-x (HBx), leading to hepatitis. If hepatitis becomes chronic, it may progress to cirrhosis or hepatocellular carcinoma. Therefore, research and development of prophylactic and therapeutic agents against HBV, which is a cause of hepatocellular carcinoma (HCC), is desirable [2]. Three HBV proteins, HBx protein, hepatitis B virus (HBs) protein, and hepatitis B virus c (HBc) protein antigens, are known to interact with mitochondria. The C-terminal truncation of HBx is caused by cellular stress, which promotes the production of ROS and impairs mitochondrial function, thus playing a role in HCC development [3,4,5,6,7].

The Nrf2 (nuclear factor-erythroid-2-related factor)–ARE (antioxidant response element) signaling pathway responds to cell damage with the excessive production of ROS and reactive nitrogen species (RNS) or electrophiles [8]. HBV-induced oxidative stress is accompanied by activation of the Nrf2/ARE pathway, leading to suppressed viral replication [9,10,11,12].

HBx contributes to HBV replication by increasing the activity of the core promoter region (RNA transcriptional regulation), which is critical for HBV replication. A recent study has shown that Nrf2 interacts with the HBV core promoter and strongly suppresses HBV core promoter activity, thereby reducing viral replication. Thus, activation of the Nrf2/ARE pathway is important for suppressing oxidative stress and inhibiting HBV replication [13].

Our previous studies have shown that marine natural products and edible plants modulate the HO-1 protein expression by activating Nrf2 in normal and cancer cells [14,15,16,17,18]. The marine carotenoid fucoxanthin (Fx), as an Nrf2 activator, activates the Nrf2–ARE pathway at low concentrations and induces the antioxidant protein HO-1, which is cytoprotective. In this study, we focused on the Nrf2 signaling under oxidative stress in HBV-infected cells and described the anti-HBV infection effects of the Nrf2 activators.

## 2. Results

### 2.1. Nrf2 Activators in This Study

**Figure 1:**
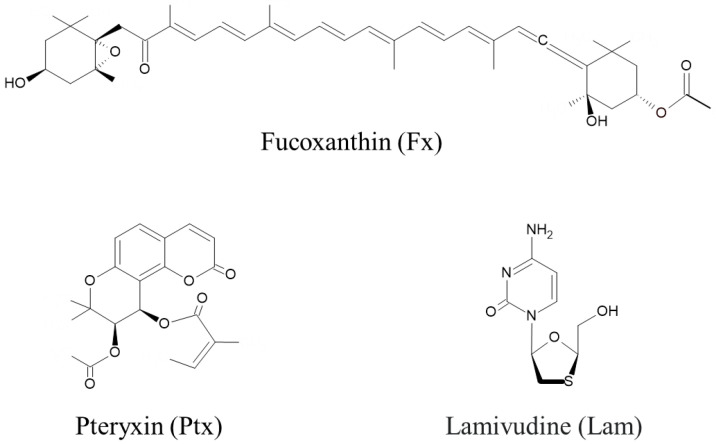
Nrf2 activators of fucoxanthin (Fx) and pteryxin (Ptx), and the hepatitis B virus (HBV) therapeutic lamivudine (Lam), were used in this study.

Figure 1 shows the chemical structures of the Nrf2 activators, fucoxanthin (Fx), pteryxin (Ptx), and the HBV therapeutic lamivudine (Lam) as a reverse transcriptase inhibitor. The major marine carotenoid Fx is found in the brown alga *Cladosiphon okamuranus*. Previous studies have reported that Fx has an Nrf2 activity, resulting in the cytoprotective effects of an antioxidant, anti-inflammatory activities, and anti-cancer activity with apoptotic induction [14,17,19,20]. Ptx is an Nrf2 activator isolated from *Peucedanum japonicum* Thunb., a well-known medicinal herb in Asia, which has the potential to prohibit cellular damage related to the expression of antioxidant genes and enzymes on the ARE region of the nuclei of insulinoma cells [16,18].

### 2.2. HBV-Infected Cells Under Oxidative Stress

**Figure 2:**
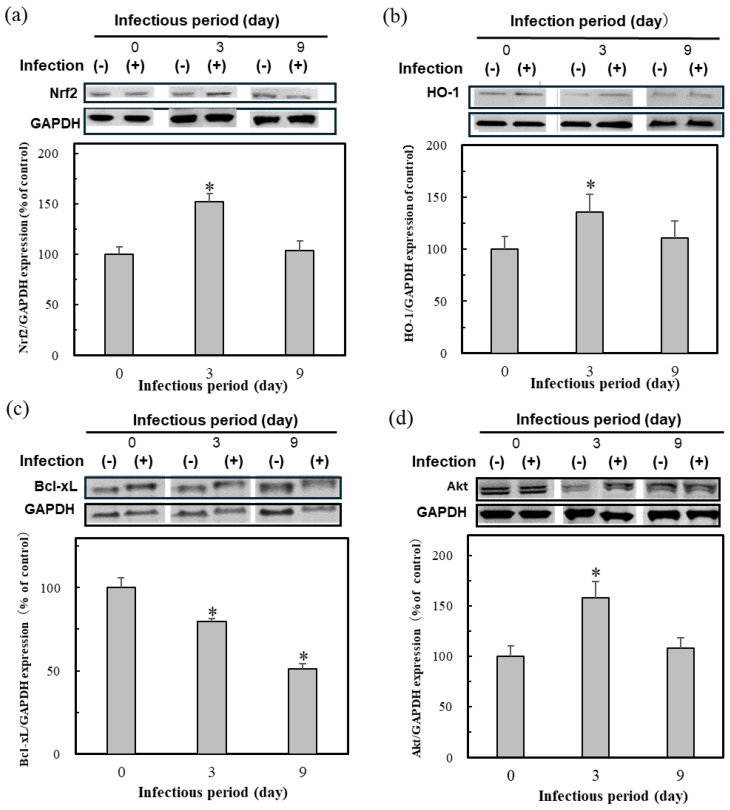
The protein expression of Nrf2 (**a**), HO-1 (**b**), Akt (**c**), and Bcl-xL (**d**) in the HBV-infected cells. Cell lysates were prepared at day 0, 3, and 9 after HBV infection in HepG2-hNTCP-C4 cells, followed by Western blotting. The ratios of the band intensities of the cellular proteins indicated to those of GAPDH were quantified and are shown at the bottom of the individual blots with the day 0 value as 100%. Data were expressed as mean ± SD and the significant differences from day 0 were analyzed by Student’s *t*-test. * *p* < 0.01 indicated as a significant difference from infection period day 0.

Figure 2 shows the result of protein expressions indicative of oxidative stress in the HBV-positive cells; the Nrf2 expression was high on day 3 after infection but decreased during continuous infection (Figure 2a). In addition, all protein expression was reduced after 9 days of infection. This result indicates the weakening of the resistance to oxidative stress due to persistent infection. For example, the antioxidant protein HO-1, which is expressed by the transcription factor Nrf2, showed similar results (Figure 2b). The anti-apoptotic protein Bcl-xL also decreased, suggesting that apoptosis was underway (Figure 2c). The Akt protein expression, which regulates Nrf2 and Bcl-xL, also showed similar trends to Nrf2 and HO-1 (Figure 2d).

These results suggest that HBV infection, especially persistent infection, requires an Nrf2 activator to keep the protective effect of the initial oxidative stress on infection. The present study then set and continued the research subject to test the efficacy of Nrf2 activators.

### 2.3. Anti-HBV Activity Due to Nrf2 Activators

**Figure 3:**
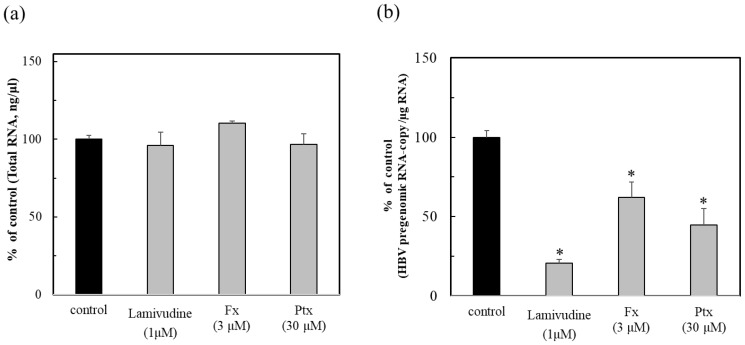
Anti-HBV activity of the Nrf2 activators fucoxanthin (Fx) and pteryxin (Ptx), and comparison with lamivudine (Lam). After culturing the HBV-producing cells (Hep38.7-Tet) with Fx (3 μM), Ptx (30 μM), and the HBV therapeutic Lam or without compound for 7 days, they were then inoculated into HepG2-hNTCP-C4 cells. The total RNA of the infected cells (**a**) were prepared at 7 days post-infection and the HBV pregenomic RNA (**b**) was measured. The anti-HBV activity of the compounds was evaluated as the % of control (without compound) (**b**). Data were expressed as mean ± SD and the significant differences were analyzed by Student’s *t*-test. * *p* < 0.01 indicated as a significant difference from the control in the HBV-infected cells.

The non-maximum toxic concentrations of the test compounds were examined for their total RNA (Supplementary Table S1). As a result, each concentration of Fx, Ptx, and Lam at 3, 30, and 1 µM was determined for the anti-HBV activity test (Figure 3a). The anti-HBV activity of Fx showed inhibition at the low concentration of 3 µM, in comparison to Ptx (at 30 μM). This result suggested that the Nrf2 activators could act effectively for persistent HBV infection on the status of infection, as shown in Figure 2. These Nrf2 activators, particularly the Fx, demonstrated a similar activity to that of the HBV polymerase inhibitor Lam (Figure 3b). This result suggested that the suppression of HBV activity by Nrf2 activity could contribute to the protection of HBV, although the functional mechanisms are different from that of the HBV therapeutic Lam. Therefore, anti-HBV agents will be considered as further targets for development as Nrf2 activators that could enhance the defensive metabolism, such as the Nrf2/ARE signaling pathway in the biological antioxidant system.

### 2.4. Activation of Nrf2 and HO-1 Due to Nrf2 Activators in HBV-Positive Cells

**Figure 4:**
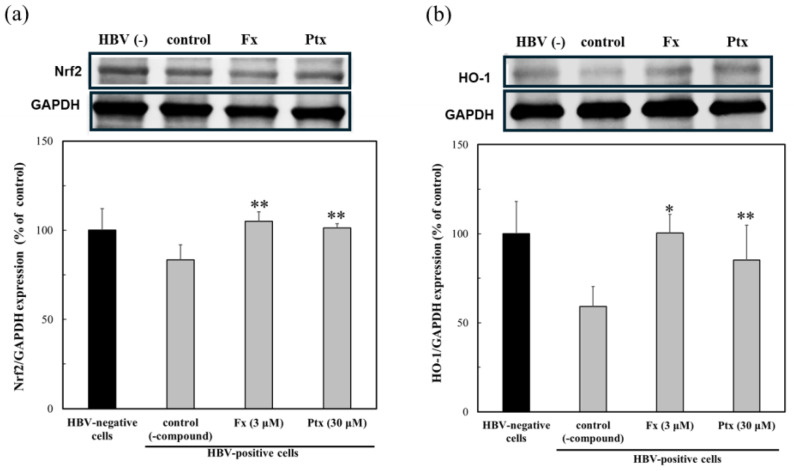
Modulation of the protein expression due to the Nrf2 activators, fucoxanthin (Fx) and pteryxin (Ptx), in HBV-infected cells. The protein expression of Nrf2 (**a**) and HO-1 (**b**) in HBV-infected HepG2-hNTCP-C4 cells in the presence of Fx (3 μM) and Ptx (30 μM) for 7 days was analyzed by Western blotting. Data were expressed as mean ± SD and the significant differences from the control without compound were analyzed by Student’s *t*-test. * *p* < 0.01 and ** *p* < 0.05 indicated a significant difference from the control (-compound) in the HBV-infected cells.

To evaluate the contribution of the Nrf2 activators, the protein expression of Nrf2 and HO-1 was assessed in HBV-positive cells (Figure 4). The Nrf2 activators treated with HBV-positive cells were moderately expressed as Nrf2 in the presence of the test compounds. The expression of HO-1 was more pronounced in the presence of both Nrf2 activators. Especially Fx, even at a 10-fold lower concentration, compared to Ptx, which was expressed more effectively.

### 2.5. Modulation of Protein Expression by Nrf2 Activators in HBV Genomic Cells

**Figure 5:**
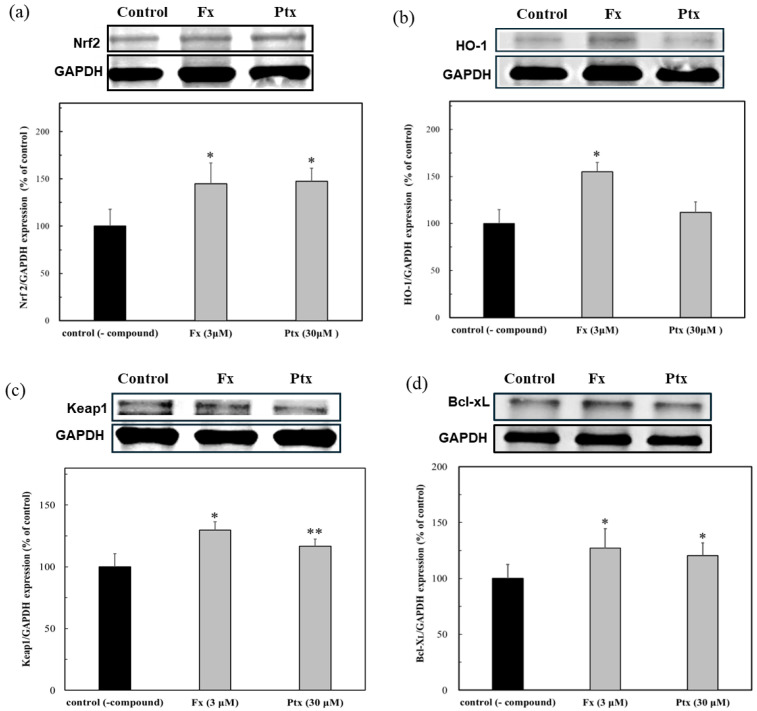
Modulation of protein expressions due to the Nrf2 activators, fucoxanthin (Fx) and pteryxin (Ptx), in HBV genome transduced cells. The proteins expression of (**a**) Nrf2, (**b**) HO-1, (**c**) Keap1, and (**d**) Bcl-xL in the presence of Fx (3 μM) and Ptx (30 μM) on the HBV-persistent cells. Data were expressed as mean ± SD and the significant differences were analyzed by Student’s *t*-test. * *p* < 0.01 and ** *p* < 0.05 indicated a significant difference from the control (-compound).

Hep38.7 cells transduced with the HBV genome were used to evaluate the anti-HBV effect of the Nrf2 activators. As shown in Figure 5a, both Nrf2 activators of Fx (3 μM) and Ptx (30 μM) activated the Ntf2 expression and, although a low concentration of Fx was used, it more effectively expressed the HO-1 protein (Figure 5b). This result demonstrated the effect of the Nrf2 activators on the persistent HBV cells. The expression of the Nrf2 and HO-1 proteins was accompanied by the induction of Keap1 expression, that is, the Nrf2 activators could be dissociated Nrf2 from Keap1, leading to the HO-1 protein expression (Figure 5c). The anti-apoptotic protein Bcl-xL in persistent cells increased (Figure 5d). The HBx protein, which is involved in HBV replication, has been shown to induce apoptosis by inhibiting Bcl-xL. This suggests that the modulation of HBx by the Nrf2 activators may have an anti-HBV effect.

## 3. Discussion

In this study, the expression of proteins related to oxidative stress in the HBV-infected cells during HBV infection was clarified (Figure 2). The HBV-infected cells protected against oxidative stress by activating the Nrf2/ARE-mediated induction of the HO-1 and the Nrf2 regulatory protein Akt. The expression of these proteins was activated during the initial infection of the 3-day infection. Previous studies have shown that HBV induces the strong activation of Nrf2/ARE-regulated genes in vitro and in vivo, suppressing the expression of the HBV replication proteins HBx and LHBs, and the generation of ROS by Nrf2-induced antioxidant enzymes [9,10,11]. Keap1 also recognizes HBx and activates the Nrf2/ARE signaling pathway during HBV infection, thereby inhibiting HBV replication [12,13]. These articles were similar to our findings of the Nrf2/ARE-mediated induction of the cytoprotective antioxidant protein HO-1 by HBV infection, resulting in the protection of the HBV-infected cells against oxidative damage. However, the protective function was reduced in persistently infected cells, suggesting that they were exposed to oxidative stress (Figure 2). Moreover, oxidative stress persisted in the infected cells for 9 days. As a result, the reduction in Akt protein expression may lead to reduction in related signaling proteins, such as the anti-apoptotic proteins Bcl-xL and Nrf2 [14,15].

These results suggested that the HBV-dependent induction of the Nrf2/ARE-regulated genes in the HBV-positive cells plays a significant role in suppressing the pathological progression of the HBV-positive cells under oxidative stress.

The lack of the HBV-dependent induction of the Nrf2/ARE-regulated genes may lead to an earlier onset of fibrosis. Based on these results, i.e., the anti-HBV activity of Fx and Ptx, the Nrf2 activators found in our previous studies were evaluated [14,17]. As shown in Figure 3, both Nrf2 activators exhibited anti-HBV activity, particularly Fx, which inhibited the HBV activity at lower concentrations than Ptx, which inhibited HBV activity similarly to that of the reverse transcriptase inhibitor Lama. Both Nrf2 activators activated HO-1 protein expression in the HBV-infected cells (Figure 4). In addition, both Nrf2 activators enhanced Nrf2, HO-1, and Keap1 expression even in the HBV-persistent cells, with the HO-1 expression being more clearly demonstrated by Fx than in the HBV-positive cells already described (Figure 5). Fx and Ptx are electrophilic and interact with Keap1 to activate the Nrf2. These Nrf2 activators also activate the Keap1 protein during HBV infection, suggesting an interaction with HBx, leading to the inhibition of HBV replication via the activation of Nrf2 and the suppression of HBV core promoter activity [13]. The HBV-dependent Nrf2 activation induces the expression of cytoprotective genes with ARE sequences in their promoters, thus reducing ROS through the expression of antioxidant enzymes [11]. These Nrf2 activators have a peroxyl radical scavenging activity involved in lipid peroxidation, and antioxidant enzyme activation via the Nrf2–ARE signaling activity; moreover, antioxidant activity via the Nrf2–ARE signaling activity may contribute to the anti-HBV activity [14,16,17,18]. In addition, the Nrf2 activators activated the anti-apoptotic protein Bcl-xL in persistent cells (Figure 5d). Recent studies have shown that HBx inhibits Bcl-xL expression and subsequently promotes cytochrome C release from mitochondria, causing the loss of the mitochondrial membrane potential and inducing apoptosis. HBx binds to the anti-apoptotic proteins Bcl-2 and Bcl-xL via a BH3-like motif. The Bcl-xL expression inhibits HBx-induced apoptosis [21,22]. Therefore, the HBx protein expression is suppressed in the presence of the Nrf2 activator, suggesting that the Nrf2 activator contributes to the protection of the HBV-infected cells by inhibiting the HBV replication protein HBx through Nrf2 activation. Further studies to elucidate the mechanism of action will be undertaken in future studies.

A summary of these results indicated that the Nrf2 activator will be useful to prohibit the replication of HBV-infected cells or delay the proceeding pathogenesis for HBV-persistent cells. In addition, the Nrf2 functions may contribute to the protection of the HBV-infected cells by inhibiting the HBV replication protein HBx.

## 4. Materials and Methods

### 4.1. Materials

Fucoxanthin (Fx) from *Nemacystus decipiens* was donated from South Product, Ltd. (Okinawa, Japan). Pteryxin was isolated based on previous procedures [16]. The other chemicals were used for the height analysis. The protein expression reagents for the SDS-page electrophoresis were purchased from Thermo Fisher Scientific (Thermo Fisher Scientific Inc., Waltham, MA, USA). The Western blot analysis reagents, EzBlock Chemi and EzWestLumi plus, were purchased from ATTO (ATTO Corporation, Tokyo, Japan). The various antibodies, such as antiNrf2, anti-Bcl-xL, and secondary antibody HRP-labeled IgG from Abcam (Abcam Limited., Cambridge, UK), and the other antibodies of anti-HO-1, anti-Akt and GAPDH, were obtained from Cell Signaling Technology (Cell Signaling Technology, Inc., Danvers, MA, USA).

### 4.2. Cell Culture

Human hepatoma HepG2-hNTCP-C4 (HBV-susceptible cells) [23] and Hep38.7-Tet cells (HBV genome-integrated cells) [24] were kindly provided by Dr. Koichi Watashi (National Institute of Infectious Diseases). These cells were maintained in Dulbecco’s modified Eagle medium (Fujifilm Wako Pure Chemical, Osaka, Japan) supplemented with 10% fetal bovine serum.

### 4.3. Virus Infection

The culture supernatants of the Hep38.7-Tet cells were collected and precleared for the preparation of the inoculum. The HepG2-hNTCP-C4 cells were pretreated overnight with a 2% DMSO (Fujifilm Wako Pure Chemical)-containing medium, and then inoculated with HBV samples containing 2% DMSO and 5% PEG8000 (Promega, Madison, WI, USA). At 48 h after infection, the culture medium was changed to a fresh 2% DMSO-containing medium to continue the culture.

### 4.4. Western Blot Analysis

The lysis cell samples were separated by SDS-page electrophoresis. The proteins were detected by a Western blot analysis using various antibodies. The protein expression was quantified by densitometry (LuminoGraph I, ATTO Corporation, Tokyo, Japan). The expression level of each protein was indicated as a percent of the GAPDH expression. The protein expression rates during the infection process are expressed as the expression level of infected cells relative to uninfected cells for four independent experiments.

### 4.5. RNA Extraction and Quantitative RT-PCR

The total RNA was extracted from transfected cells using TRI reagent (Molecular Research Center, Cincinnati, OH, USA) according to the manufacturer’s instructions. Quantification of the HBV RNA and host-derived mRNA was performed as previously described [25]. The primer set used for the HBV pregenomic RNA was F: TCCCTCGCCTCGCAGACG and R: GTTTCCCACCTTATGAGTC.

### 4.6. Evaluation of Anti-HBV Activity of Nrf2 Activators

The HBV-producing cells (Hep38.7-Tet) were cultured with Fx (3 μM) and Ptx (30 μM) or without compound for 7 days, and their culture supernatants were diluted 5-fold and inoculated into the HepG2-hNTCP-C4 cells. The total RNA of the infected cells were prepared at 7 days post-infection, and the HBV pregenomic RNA was measured by quantitative RT-PCR. The highest concentration of non-toxic test compound (no effect on the total RNA content) was evaluated for anti-HBV activity (Supplementary Table S1). The anti-HBV activity of the compounds was performed in triplicate independent experiments and expressed as HBV pregenomic RNA copy per 1 µg of RNA.

### 4.7. Modulation of the Protein Expressions Due to Nrf2 Activators in the HBV Genome-Transduced Cells

The modulation of the protein expressions due to the Nrf2 activators, Fx (3 μM) and Ptx (30 μM), in the Hep38.7-Tet cells (HBV genome-integrated cells) for four independent experiments were incubated for 6 days. The protein expression was detected by a Western blot analysis, as described in Section 4.4.

### 4.8. Statistical Analysis

The results were indicated as mean ± SD and the significant differences were expressed as * *p*< 0.01 and ** *p* < 0.05.

## 5. Conclusions

This study was conducted to elucidate the involvement of oxidative stress in HBV-infected cells and the efficacy of natural Nrf2 activators to protect against oxidative stress-induced cell damage with excessive ROS production. The results showed that the oxidative stress-related proteins HO-1, Akt, and Bcl-xL were decreased by continuous infection, indicating that HBV-positive cells were exposed to oxidative stress. In addition, this study showed that the Nrf2 activators, Fx and Ptx, suppressed the HBV pregenomic RNA production in the HBV-infected cells, which has a potential protective effect on the progressive pathogenesis of HBV through the activation of Nrf2. In the persistently infected cells transfected with the HBV genome, the proteins Bcl-xL and Keap1, which contribute to the suppression of the HBx protein involved in HBV replication, were overexpressed. In particular, Fx showed remarkable protein expression at low concentrations. Therefore, our present study demonstrated that Nrf2 activation may be a potential target for the development of host defense strategies against HBV infection.

## Data Availability

All original data are available from the corresponding author upon request.

## Supplementary Materials

The following supporting information can be downloaded at: https://www.mdpi.com/article/10.3390/md23040155/s1, Figure S1: The cell assay protocol for anti-HBV activity due to Nrf2 activators; Table S1: The results of this preliminary experiment for anti-HBV activity.
