# Supplementary material for: Effect of Nrf2 Activators in Hepatitis B Virus-Infected Cells Under Oxidative Stress
Source: Mar Drugs. 2025 Apr 3;23(4):155. doi: 10.3390/md23040155 (PMC12028886; doi:10.3390/md23040155)
Supplement: Supplementary file 1 [file marinedrugs-23-00155-s001.zip › Supplementary Figure1.pptx]

## Slide 1
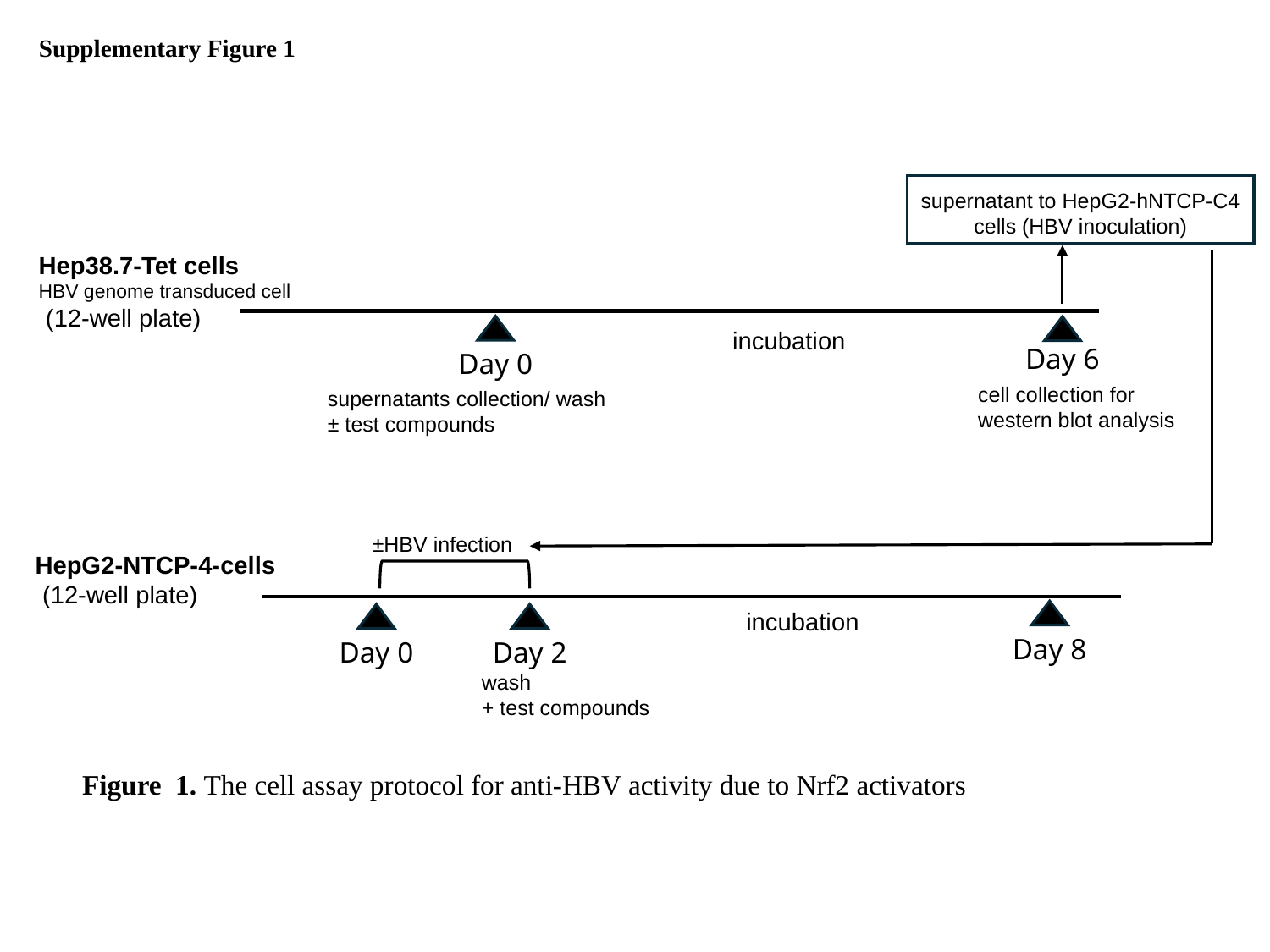

Supplementary Figure 1
supernatant to HepG2-hNTCP-C4 cells (HBV inoculation)
Hep38.7-Tet cells
HBV genome transduced cell
 (12-well plate)
incubation
Day 6
Day 0
cell collection for western blot analysis
supernatants collection/ wash
± test compounds
±HBV infection
HepG2-NTCP-4-cells
 (12-well plate)
incubation
Day 8
Day 0
Day 2
wash
+ test compounds
Figure 1. The cell assay protocol for anti-HBV activity due to Nrf2 activators
