# Supplementary material for: Effect of Nrf2 Activators in Hepatitis B Virus-Infected Cells Under Oxidative Stress
Source: Mar Drugs. 2025 Apr 3;23(4):155. doi: 10.3390/md23040155 (PMC12028886; doi:10.3390/md23040155)
Supplement: Supplementary file 1 [file marinedrugs-23-00155-s001.zip › Supplementary Table 1.pptx]

## Slide 1
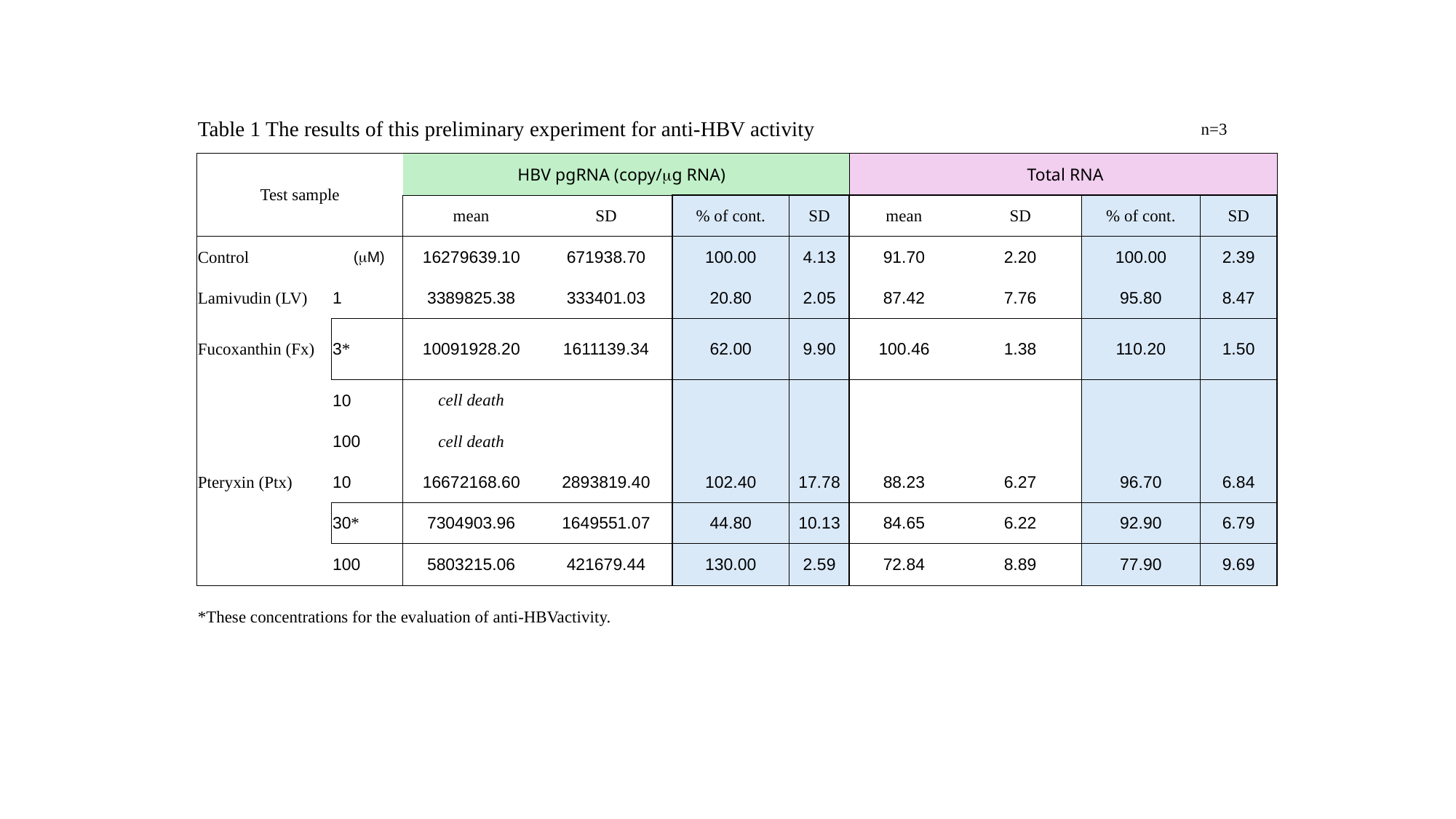

| Table 1 The results of this preliminary experiment for anti-HBV activity | | | | | | | | | n=3 |
| --- | --- | --- | --- | --- | --- | --- | --- | --- | --- |
| Test sample | | HBV pgRNA (copy/mg RNA) | | | | Total RNA | | | |
| | | mean | SD | % of cont. | SD | mean | SD | % of cont. | SD |
| Control | (mM) | 16279639.10 | 671938.70 | 100.00 | 4.13 | 91.70 | 2.20 | 100.00 | 2.39 |
| Lamivudin (LV) | 1 | 3389825.38 | 333401.03 | 20.80 | 2.05 | 87.42 | 7.76 | 95.80 | 8.47 |
| Fucoxanthin (Fx) | 3\* | 10091928.20 | 1611139.34 | 62.00 | 9.90 | 100.46 | 1.38 | 110.20 | 1.50 |
| | 10 | cell death | | | | | | | |
| | 100 | cell death | | | | | | | |
| Pteryxin (Ptx) | 10 | 16672168.60 | 2893819.40 | 102.40 | 17.78 | 88.23 | 6.27 | 96.70 | 6.84 |
| | 30\* | 7304903.96 | 1649551.07 | 44.80 | 10.13 | 84.65 | 6.22 | 92.90 | 6.79 |
| | 100 | 5803215.06 | 421679.44 | 130.00 | 2.59 | 72.84 | 8.89 | 77.90 | 9.69 |
| \*These concentrations for the evaluation of anti-HBVactivity. | | | | | | | | | |
